# The Idea Is Mine! An Empirical Examination on the Effect of Leaders’ Credit Claiming on Employees’ Work Outcomes

**DOI:** 10.3389/fpsyg.2022.818454

**Published:** 2022-02-18

**Authors:** Siyuan Chen, Daiheng Li, Chun Yang, Xijing Zhang, Liang Hou

**Affiliations:** ^1^School of Economics and Management, Beijing Jiaotong University, Beijing, China; ^2^Business School, Beijing Wuzi University, Beijing, China; ^3^School of Business, Renmin University of China, Beijing, China

**Keywords:** credit claiming, anger, perceived unfairness, work outcomes, affective events, relative deprivation

## Abstract

Existing studies mainly explored the detrimental effect of employee credit claiming, and little is known about how leader credit claiming can affect employees. Based on affective events theory and relative deprivation theory, we explore how leader credit claiming affects employee work outcomes (i.e., voice behavior and job performance) by the research methods of literature review, interview, and empirical questionnaire. With a sample of 418 matched leader–employee pairs from a large manufacturing company, we find that leader credit claiming influences employee work outcomes through the mediating role of employee anger and perceived unfairness. Additionally, we determine that leader credit-claiming attribution (i.e., to protect employees) has a moderating influence on the relationship between credit claiming and anger and between credit claiming and perceived unfairness. The results support all hypotheses. Furthermore, we discuss the theoretical and practical implications of the findings.

## Introduction

Credit claiming is defined as an individual’s appropriation of other individuals’ contributions in an organization or exaggerating one’s role in an event to present a positive work image to supervisors (Weaver, 1986; Ellis et al., 2002). Credit claiming is a common phenomenon in the workplace and has a detrimental effect on other employees’ performance, emotional commitment, and relationships with colleagues (Dutton and Ashford, 1993; Grant et al., 2009; Hendriks et al., 2020). Given its harmful effects (Xu et al., 2019), considerable research was conducted to understand further the nature and impact of credit claiming. Some studies identified the negative effect of credit claiming on other employees’ psychological wellbeing (Glazer and Segendorff, 2005; Rupp et al., 2017). Meanwhile, other studies explored the relationship between credit claiming and the outcomes of other employees and found that credit claiming can hinder other employees’ work engagement and service performance (Liao and Chuang, 2004; Arazy and Gellatly, 2012).

Despite the advancements in studies on credit claiming, previous research on the topic can be expanded in several ways. First, the phenomenon of leaders’ appropriation of employees’ contributions and claiming credit to impress senior managers were rarely examined (Reichheld, 1993; Proell et al., 2016; Abrahms and Conrad, 2017). Although some studies explored the impact of employees’ credit claiming on coworkers’ attitudes and behaviors, leaders are more willing to claim credit for employees’ superior performance and competence than other employees owing to the role expectations of organizational stakeholders (Maher et al., 2018; Brosy et al., 2021). Leaders also have power over their employees to claim credit for their actions, even when infringing on their employees’ interests (Nevicka et al., 2018; Qian et al., 2018). However, in such cases, employees dare not speak up. Thus, examining the significance of leaders’ credit-claiming behavior is essential. Second, though the credit-claiming phenomenon was explored from the perspective of resource conservation (Gerber and Gibson, 2009; Deng et al., 2018; Xia et al., 2019), little research was conducted on how credit claiming affects employees’ emotions and behaviors in terms of affective events and deprivation. In addition, the role of the cognitive attribution of individuals who take the benefits of credit claiming remains underexplored (Clapham and Schwenk, 1991; Vogelgesang et al., 2021). Employees’ use of attributional information can make different judgments about their leaders’ credit-claiming behavior, which can generate different outcomes. Therefore, this study proposes the below mentioned three research questions (RQ):

RQ1. Does leaders’ credit claiming affect employees’ work outcomes in the organization?
RQ2. How does leaders’ credit claiming affect employees in terms of affective events and deprivation?
RQ3. Does the cognitive attribution affect the relationship between leaders’ credit claiming and employees’ emotions?

**FIGURE 1:**
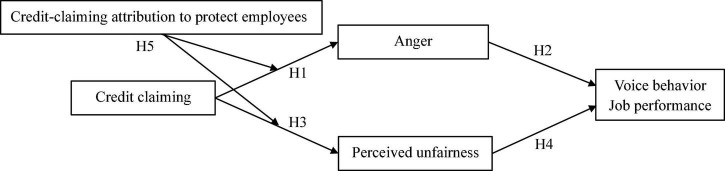
Conceptual model.

To fill the research gaps and examine the effect of leaders’ credit claiming on employees’ work outcomes, this study establishes a model based on affective events theory and relative deprivation theory (Erdogan et al., 2018; Zhou et al., 2018; Crawford et al., 2019; Lee et al., 2021). According to this model, leaders’ credit claiming can induce employees to feel deprived by their leaders, leading to anger and psychological perceptions of unfairness. Such negative emotions and psychological states can affect employees’ subsequent work outcomes (i.e., voice behavior and job performance). In addition, the impact of leaders’ credit-claiming behavior on employees may differ depending on the employees’ attribution of their leaders’ actions (i.e., to protect employees). For example, if employees attribute their leaders’ credit claiming of their suggestions to their leaders’ desire to protect them, then the perceived risk transfer of the suggestions may mitigate the extent of the negative impact of the leaders’ credit claiming (Morrison, 2011). Therefore, this study examines credit-claiming attribution as a moderator in the relationship between leaders’ credit claiming and employees’ anger and between leaders’ credit claiming and employees’ perceived unfairness. Figure 1 displays our theoretical framework.

The novelty and contributions of this study reflect in the following three points. First, this study contributes to research on credit claiming by illustrating the negative effect of leaders’ credit claiming on employees’ work outcomes. Leaders who deprive employees of their contributions can anger the employees, who would not dare speak up, triggering poor work outcomes (Vigoda, 2000; De Clercq et al., 2019). In other words, in most cases, leaders’ credit-claiming behavior may also be detrimental to an organization’s operations and development (Deshpande, 1997). Therefore, examining the consequences of leaders’ credit-claiming behavior is essential. Second, this study extends affective events theory and relative deprivation theory by revealing the mediating role of employees’ anger and perceived unfairness. This research provides a new theoretical perspective and explains how leaders’ credit claiming can affect employees’ work outcomes through anger and perceived unfairness. Third, this study determines how and under what conditions leaders’ credit claiming can affect employees’ emotional and psychological states by elucidating employees’ perception of credit-claiming attribution. Furthermore, this research enriches the literature on credit claiming by exploring the downstream effect of leaders’ credit claiming on employees.

The structure of this study is as follows (Rasool et al., 2021; Wang et al., 2021). In the “Theoretical development and hypotheses” section, the theoretical development and hypotheses are briefly discussed. The research methods are explained in the “Methods” section. The results and analysis are presented in the “Results” section. The discussion of this study presents in the “Discussion” section. The “Conclusion” section discusses the conclusions of this study.

## Theoretical Development and Hypotheses

### Affective Events Theory

Affective events theory focuses on the structure, triggers, and consequences of employees’ affective reactions in the workplace and suggests that stable work environment features can lead to positive or negative work events (Weiss and Cropanzano, 1996; Pirola-Merlo et al., 2002). Experiencing such work events can trigger an affective response, influencing employees’ attitudes and behaviors (Cropanzano et al., 2017b). According to affective events theory, a particular work event can exert a positive or negative impact on an employee’s wellbeing or other aspects (Williams et al., 1991; Weiss and Cropanzano, 1996; Gaddis et al., 2004; Beal et al., 2005). Such events may trigger affective reactions in employees (Weiss and Cropanzano, 1996). When an affective event occurs, an employee will react to the event with a series of emotional responses, including assessing the affective event and other environmental factors and feeling specific emotions (Cropanzano et al., 2017b).

After its introduction, affective events theory garnered attention from the academic community. Under the guidance of its principles, various studies tested and validated the theory from different perspectives. Some studies treated leaders’ work behaviors as affective events that influence employees’ attitudes and behaviors (Judge et al., 2017; Jiang and Lavaysse, 2018) and explored the role of leaders’ attitudes and behaviors in influencing employees (Inceoglu et al., 2018; Oreg and Berson, 2019). Gaddis et al. (2004) found that negative feedback from leaders to employees can trigger employees’ affective reactions, harming how employees evaluate their leaders’ performance and their team’s quality. Cropanzano et al. (2017b) investigated the role of emotions in establishing exchange relationships between leaders and employees and found that emotions play a crucial role in relationship formation, establishment, and routinization (Cropanzano et al., 2017b).

### Relative Deprivation Theory

Relative deprivation theory describes how individuals’ particular subjective states can influence their emotions, cognitions, and behaviors (Crosby, 1976; Smith et al., 2012). According to Smith et al. (2012), relative deprivation theory consists of three parts. First, individuals compare themselves with others, including members of the same group, different groups, and outsiders. Second, individuals make cognitive evaluations and identify the disadvantages of their groups. Third, individuals’ perception of disadvantages can induce a sense of unfairness, leading to feelings of anger.

Based on relative deprivation theory, individuals become angry and resentful when they compare themselves with other people, groups, or themselves and perceive that they are not getting what they deserve (Erdogan et al., 2018; Xiong et al., 2021). Moreover, relative deprivation theory states that when individuals compare themselves with other members of a group in terms of something they care about, they will experience a range of emotions if they find themselves at a disadvantage (Yang et al., 2021), including a sense of injustice and that they deserve better or anger and hatred toward the factors that led to their disadvantaged position (Jung et al., 2018). For example, when employees compare themselves with other team members and find that their colleagues receive a higher salary for the same work, they may feel a sense of unfairness. In addition, they may feel strongly that they deserve the high salary and anger toward their colleagues or leaders causing the unfair treatment.

### Credit Claiming, Anger, and Work Outcomes

Leaders’ credit claiming refers to the use of dogmatism to deprive employees of their contributions or the exaggeration of their role in an event to impress senior managers (Weaver, 1986; Ellis et al., 2002). From employees’ perspective, the behavior involves leaders claiming credit for their ideas, opinions, or suggestions to impress senior leadership (Ellis et al., 2002). For example, if an employee’s proactive behavior (e.g., creative behavior) results in a positive change in the organization (e.g., improved performance), the positive change is within the leader’s area of responsibility. The leader may claim credit for the employee’s contribution to the senior leadership team (Fuller et al., 2015).

Leaders are motivated to manage impressions to create a positive image of their superior performance and capabilities owing to the responsibilities accompanying their position and stakeholders’ expectations (e.g., senior managers, shareholders, and customers), such as to improve company productivity and facilitate organizational changes (Maher et al., 2018). Leaders also manage impressions owing to the power given to them by their position (Brosy et al., 2021). Specifically, leaders have power over their employees (e.g., the right to evaluate their performance and assign tasks); thus, when they infringe on their employees’ interests, the employees may become angry but will not dare speak out (Nevicka et al., 2018; Qian et al., 2018). In addition, the nature of the relationship between leaders and their subordinates makes delineating and verifying specific merits and contributions difficult (Dunegan et al., 1992).

According to affective events theory, when a leader claims credit for an employee’s contribution and uses it to gain credit from upper management, the employee will perform a cognitive analysis of the affective event, which will trigger an emotional response (Weiss and Cropanzano, 1996; Pirola-Merlo et al., 2002; Cropanzano et al., 2017b). Leaders’ credit-claiming behavior violates employees’ interests. Employees analyze and interpret affective events according to different scenarios and personalities (Humphrey, 2002). Thus, this study suggests that leaders’ credit-claiming behavior violating employees’ interests triggers negative emotions in the employees. Specifically, this research focuses on employees’ feelings of anger. According to the theory of relative deprivation, when a leader claims credit for an employee’s contribution, the employee will perceive being at a disadvantage (Crosby, 1976; Smith et al., 2012). Employees may feel that their leaders take their hard work or ideas without effort. This perception of their disadvantageous position may trigger anger in employees.

Employees’ anger can be defined as unpleasant solid, or hostile feelings of intentional and controllable harm (Douglas and Martinko, 2001; Detert et al., 2007). When employees observe leaders’ credit-claiming behavior, they may become angry for several reasons. First, leaders’ appropriation of employees’ contributions violates the latter’s established interests, including their sense of responsibility by contributing to the organization and satisfaction of their accomplishments, proving their abilities and value. Second, leaders’ credit-claiming behavior is intentional (Ellis et al., 2002). Although leaders are aware of the unethical nature of credit claiming, they are driven to engage in the behavior by their power, pressure from their role, and the convenience of the nature of their relationship with their employees (Nevicka et al., 2018; Brosy et al., 2021). Third, leaders’ credit claiming of employees’ contributions is controllable (Ellis et al., 2002). Leaders can choose other ways to manage impressions and prove their competence (Turnley and Bolino, 2001). Based on the above analysis and discussion, this study argues that when employees are confronted with their leaders’ deprivation of their contributions and credit claiming, they will analyze the affective event and feel anger. Thus, the following hypothesis is proposed:

*Hypothesis 1: Leaders’ credit claiming is positively related to employees’ anger.*

Moreover, this study argues that employees’ emotional reactions to their leaders’ credit-claiming behavior impact their subsequent work outcomes. This research focuses on employees’ work outcomes, including in-role (i.e., job performance) and extra-role (i.e., voice behavior) behaviors. According to affective events theory, individuals’ behaviors are influenced by not only rational factors but also the intervention of their emotional reactions (Gaddis et al., 2004; Oreg and Berson, 2019). When an event violates an individual’s valued rights, it will trigger a negative emotional response (e.g., anger or sadness). The theory further clarifies that individuals’ emotional responses to an event take precedence over other behaviors (Cropanzano et al., 2017b; Inceoglu et al., 2018). Taylor (1991) observed that adverse events prompt individuals to adjust and adapt mentally, cognitively, emotionally, and behaviorally. After the disappearance of an adverse affective event, individuals may continue engaging in emotional repair and attempt to diminish the adverse effects of the affective event (Taylor, 1991).

This study argues that employees’ feelings of anger have a negative effect on their voice behavior and job performance. Anger is a high-intensity negative emotion that can induce strong emotional reactions in employees (Dasborough, 2006). According to affective events theory, to calm their anger, employees must engage in a series of adjustment behaviors (Weiss and Cropanzano, 1996). Such behaviors can significantly deplete employees’ internal resources and negatively affect other outcomes, such as voice behavior and work task completion (Byrne et al., 2014). Previous research found that employees’ negative emotions have a negative impact on their voice behavior (Hsiung and Tsai, 2017) and job performance (Sy et al., 2006). The above discussion leads to the following hypotheses:

*Hypothesis 2: Employees’ anger is negatively related to their voice behavior (H2a) and job performance (H2b).*

### Credit Claiming, Perceived Unfairness, and Work Outcomes

Perceived unfairness refers to employees’ perception of how unfairly they are treated in the workplace (Rupp et al., 2018). According to relative deprivation theory, perceptions of unfairness arise when employees compare themselves with colleagues and find themselves at a disadvantage (Smith et al., 2012; Erdogan et al., 2018). That is, employees find themselves at a disadvantage when they observe their leaders depriving them of their ideas or creativity to claim credit for and reap the benefits of their contributions from senior managers (Xiong et al., 2021). Moreover, employees may perceive that the organization does not recognize their contributions and efforts and that their leaders can easily reap the outcomes for themselves. This assessment of their disadvantage can induce a sense of unfairness in employees.

Employees’ perception of fairness derives from whether they are treated fairly in their interpersonal interactions with leaders, and leaders must adequately explain and justify the procedures performed or outcomes assigned. The hidden meaning behind leaders’ credit-claiming behavior is taking employees’ contributions for themselves without informing the employees or their consent (Weaver, 1986). This type of forced deprivation denies employees the proper and reasonable treatment for their contributions (Mauno et al., 2005). In addition, leaders’ credit-claiming behavior indicates that they have yet to adequately explain and justify their employees’ contributions (Weaver, 1986; Ellis et al., 2002). Employees may perceive that they are not receiving adequate benefits for their time and effort, whereas their leaders enjoy all the fruits of their labor. The difference between what employees put in and what they get in return can leave a huge gap. This disparity can induce feelings of lack of organizational equity in employees. Therefore, based on the analysis of relative deprivation theory and the unfairness mechanism in the process of leaders’ credit claiming, this study supposes that leaders’ credit-claiming behavior may induce feelings of unfairness in employees. The above discussion is summarized in the following hypothesis:

*Hypothesis 3: Leaders’ credit claiming is positively related to employees’ perceived unfairness.*

This study argues that employees’ perception of unfairness has a negative effect on their voice behavior and job performance. According to social exchange theory, when employees perceive unfairness in an organization, they may lack a sense of obligation and responsibility to give back to the firm (Konovsky and Pugh, 1994; Cropanzano et al., 2017a). Owing to their lack of sense of obligation and responsibility, employees will not engage in behaviors that will help the organization operate efficiently (Konovsky and Pugh, 1994), including performing their job efficiently, improving their performance, and engaging in extra-role behaviors (i.e., voice behavior) that can benefit the organization.

Based on the characteristics of organizational inequity, when employees perceive organizational unfairness, they may perceive being treated unfairly and receiving an unfair share of the outcomes (Moorman, 1991). Perceptions of unfairness can further discourage employees from engaging in behaviors (i.e., voice behavior and job performance) that can benefit the organization (Ciobanu et al., 2019). Previous research showed that employees’ perceived unfairness has a negative impact on their voice behavior (Bies and Shapiro, 1988) and job performance (Walumbwa et al., 2009). To summarize the above discussion, the following hypotheses are proposed:

*Hypothesis 4: Employees’ perceived unfairness is negatively related to their voice behavior (H4a) and job performance (H4b).*

### Moderating Effect of Credit-Claiming Attribution

When an event occurs, individuals will analyze and interpret the reasons for its occurrence (Kelley and Michela, 1980). In organizational research, individuals’ essential cognitive judgment of an event’s causes, development, and effects is referred to as attribution processes (Bulman and Wortman, 1977; Dahlin et al., 2018). Attribution processes can help employees clearly understand and control their surroundings and increase their problem-solving effectiveness (Repenning and Sterman, 2002). In this study, leaders’ credit-claiming behavior is attributed to their desire to protect their employees. Employees who voice their intention of changing the organization’s status quo or point out existing problems pose a risk to leaders (Maynes and Podsakoff, 2014). The organization may perceive such employees as troublemakers and thus disadvantaged in subsequent job assignments and performance evaluations (Burris et al., 2013). At this point, by claiming credit for their employees’ actions, leaders shoulder the potential risks and share the possible adverse effects of their employees’ voice behavior.

Based on the specific connotation of attribution, this research proposes that attribution has a moderating effect on the relationship between leaders’ credit claiming and employees’ anger and between leaders’ credit claiming and employees’ perceived unfairness. In an attribution situation, leaders claim credit to protect their employees, thereby allowing them to focus on the potential risks associated with their actions. Risks are transferred to the leaders as a result of their credit claiming. This perception of risk transfer prompts employees to appreciate the protection and support of their leaders (Detert and Burris, 2007). In this attributional state, the positive relationship between leaders’ credit claiming and employees’ anger is weakened, and the positive relationship between leaders’ credit claiming and employees’ perceived unfairness. The above discussion is summarized in the following hypotheses:

*Hypothesis 5: Leaders’ credit-claiming attribution to protect their employees may weaken the positive effect of leaders’ credit claiming on employees’ anger (H5a) and perceived unfairness (H5b).*

## Materials and Methods

### Research Approach

The survey approach, based on an empirical questionnaire, was adopted in this research. The questionnaire design and data collection were based on the hypotheses above and started with the help of a quantitative method that was followed by a descriptive or inferential application. Questionnaire surveys are a popular and extensively used research technique for the quick collection and analysis of data from a target population (Rasool et al., 2019; Samma et al., 2020).

### Sample and Procedure

The participants of this study were the employees and leaders of a large manufacturing company (Shanghai STEP Electric Corporation) in China. Before conducting the survey, we interviewed employees who perceived their leaders’ credit claiming in the company as common and therefore appropriate for this research. The respondents included 157 leaders and 646 employees who volunteered to participate in the study and were assured anonymity and confidentiality. With a list provided by the organization’s human resource department, we used a matching four-digit code to identify each leader and employee.

To reduce potential common method bias, we collected three waves of data, with each wave separated by 1 month. Some researchers have shown that using multiple waves of data collection can significantly reduce the common method bias (Podsakoff et al., 2003; Malhotra et al., 2006). At Time 1, we asked the employees about their leaders’ credit-claiming behavior and credit-claiming attribution to protect them and the control variables. At Time 2, we asked the employees questions concerning the variables of anger and perceived unfairness. At Time 3, we asked the leaders questions related to the variables of voice behavior and job performance.

In wave 1, we distributed 646 questionnaires to the employees and received 570 completed forms. In wave 2, we distributed 570 questionnaires to the employees who submitted valid questionnaires in wave 1 and received 494 completed forms. In wave 3, we distributed questionnaires to the leaders of the 494 employees and received 132 valid forms. After eliminating the invalid questionnaires (e.g., those with the same answer for all the items and those with missing values), we obtained 418 leader–employee pairs (125 leaders and 418 employees) with valid data by matching the four-digit codes (67.62% of the initial sample).

As shown in Table 1, the majority of the participants were male (59.48% males and 40.52% females). The age groups of the participants were 20–29 years (37.75%), 30–39 years (53.59%), and 40–49 years (8.66%). In terms of their education level, the participants attained a high school degree (5.71%), junior college degree (16.02%), bachelor’s degree (56.72%), or a master’s degree (21.55%).

**TABLE 1:** Sample demographics (*N* = 543).

Demographic characteristics	Participants (%)
**Gender**	
Male	323 (59.48)
Female	220 (40.52)
**Age (years)**	
20–29	205 (37.75)
30–39	291 (53.59)
40–49	47 (8.66)
**Education**	
High school	31 (5.71)
Junior college	87 (16.02)
Bachelor’s degree	308 (56.72)
Master’s degree	117 (21.55)

### Measures

As all the measures were originally written in English, we used the back-translation method to translate all the items. We used a seven-point Likert-type scale (ranging from 1 = *completely disagree* to 7 = *completely agree*) for all the measures. A pilot study was conducted to check the reliability and validity of the questionnaire. For the pilot study, we selected twelve academic professors and twelve SME entrepreneurs (who were aware of the topic of this study) to review the questionnaire. Their feedback led to several changes in item wording and the final version of the survey. To check the face validity of respondents, the study refined the questionnaire wording, assessed logical consistencies, judged the ease of understanding, and identified areas for improvement. Overall, the questionnaire was regarded as concise and easy to complete. The revised questionnaire was distributed among the selected population. All items that we used in the questionnaire are given in Supplementary Appendix A.

#### Credit Claiming

We assessed the leaders’ credit claiming with a three-item scale from Proell et al. (2016). A sample item is “My leader uses my ideas without acknowledging that I came up with them” (α = 0.927). The items used in the study were considered valid because of their alpha value above the standard of 0.70.

#### Anger

We used a four-item scale from Ford et al. (2018) to assess the employees’ anger. A sample item is “I am mad with my leader” (α = 0.907). The standard value of alpha is 0.70 and higher. So, the items we used in this research instrument were valid.

#### Perceived Unfairness

We employed the three-item scale developed by Grover (1991) to measure the employees’ perceived unfairness. A sample item is “I feel that my leader made the wrong choice that is unfair to me” (α = 0.949). The items used in the study were considered valid because of their alpha value above the standard of 0.70.

#### Voice Behavior

The leaders evaluated their employees’ voice behavior using a four-item scale from Van Dyne and LePine (1998). A sample item is “This employee developed and made recommendations concerning issues that affect the organization” (α = 0.914). The standard value of alpha is 0.70 and higher. So, the items we used in this research instrument were valid.

#### Job Performance

The leaders evaluated their employees’ job performance using the five-item job performance scale developed by Tsui et al. (1997). A sample item is “This employee’s ability to perform job tasks” (α = 0.928). The items used in the study were considered valid because of their alpha value above the standard of 0.70.

#### Credit-Claiming Attribution to Protect Employees

We used a four-item scale from Yorges et al. (1999) to assess the leaders’ credit-claiming attribution to protect their employees. A sample item is “I perceive that my leader was acting on the basis of true beliefs to protect me in his/her credit claiming” (α = 0.953). The standard value of alpha is 0.70 and higher. So, the items we used in this research instrument were valid.

#### Control Variables

We controlled for the employees’ age, gender, and education level to rule out the possibility that the aforementioned demographics may influence the outcomes.

## Results

### Common Method Bias

We performed Harman’s single-factor test to assess the presence of common method bias. Results show that the first factor explained 31.88% of the total variance, which is less than the critical standard of 40%, and that 81.96% of the total variance was explained (Podsakoff and Organ, 1986). Thus, there was no serious common method bias in this study.

### Data Analysis

Structural equation modeling (SEM) is used to analyze the relationships in the research model, and the partial least squares (PLS)-SEM method is adopted (Muthén and Muthén, 2010). In addition, SmartPLS version 3.2.8 (SmartPLS GmbH, Bönningstedt, Germany) is employed to analyze the collected data and test the hypotheses (Preacher et al., 2006).

Although this study uses previously validated scales to measure the variables, their reliability and validity are reevaluated owing to differences in the backgrounds and participants. Cronbach’s alpha is calculated to measure the scales’ reliability. In Table 2, the value of each construct is greater than the cutoff value of 0.700; thus, the reliability is acceptable. The study used factor loadings to test convergent validity. The factor loadings of all items were higher than the suggested value of 0.500, as shown in Table 2, which showed high convergence. Composite reliability is also calculated to measure convergent validity. The convergent validity of the scales is acceptable, as all the CR values are greater than the cutoff value of 0.700, and all the AVE values are greater than the cutoff value of 0.500. Table 3 shows all the correlations between the two constructs. The discriminant validity is acceptable, as the square root of the AVE is above the correlation between each construct and the other constructs (Hair et al., 2010).

**TABLE 2:** Cronbach’s alpha, factor loadings, composite reliability, and AVE values.

Construct	Number of Items	Cronbach’s alpha	Range of factor loadings	Factor 1	Factor 2	Factor 3	Factor 4	Factor 5	Composite reliability	AVE
1. Credit claiming	3	0.927	0.918–0.949	0.944	0.918	0.949			0.956	0.878
2. Anger	4	0.907	0.872–0.896	0.879	0.896	0.890	0.872		0.935	0.782
3. Perceived unfairness	3	0.949	0.948–0.960	0.948	0.960	0.951			0.967	0.908
4. Voice behavior	4	0.914	0.861–0.882	0.873	0.861	0.879	0.882		0.928	0.764
5. Job performance	5	0.928	0.865–0.912	0.871	0.912	0.890	0.865	0.883	0.947	0.782
6. Credit-claiming attribution to protect employees	4	0.953	0.938–0.969	0.938	0.960	0.951	0.969		0.976	0.911

**TABLE 3:** Correlations between two constructs.

	1. Credit claiming	2. Anger	3. Perceived unfairness	4. Voice behavior	5. Job performance
1. Credit claiming					
2. Anger	0.447				
3. Perceived unfairness	0.342	0.248			
4. Voice behavior	–0.399	–0.395	–0.376		
5. Job performance	–0.323	–0.387	–0.362	0.419	
6. Credit-claiming attribution to protect employees	–0.061	–0.052	–0.012	0.080	0.083

### Hypothesis Testing

To identify the effect of the demographic factors on the research model, gender, age, and education level are added into the research model as control variables. As shown in Table 4, the multivariate coefficient of determination is calculated. Cohen’s *f*^2^ is used to assess the effect of the control variables (i.e., insignificant: < 0.020; small: ≥ 0.020 and < 0.150; medium: ≥ 0.150 and < 0.300; and large: ≥ 0.350). The effect size of all the control variables is insignificant.

**TABLE 4:** Results of multivariate coefficient of determination (*R*^2^).

Variables	R-squared		Control variable effect		
	With control variables	Without control variables	Δ*R*^2*a*^	*f* ^2*b*^	Effect
Anger	0.201	0.201	<0.001	<0.001	Insignificant
Perceived unfairness	0.118	0.118	<0.001	<0.001	Insignificant
Voice behavior	0.242	0.238	0.003	0.004	Insignificant
Job performance	0.241	0.237	0.003	0.004	Insignificant

*^a^ΔR^2^ : R^2^ with control variables – R^2^ without control variables; ^b^f^2^ : Cohen’s f^2^.*

Table 5 presents the magnitude and significance of the path coefficients. Specifically, credit claiming is positively related to anger (*b* = 0.439, *p* < 0.001), and anger is negatively and significantly related to voice behavior (*b* = −0.321, *p* < 0.001) and job performance (*b* = −0.306, *p* < 0.001). Thus, Hypotheses 1, 2a, and 2b are supported. Meanwhile, credit claiming is positively related to perceived unfairness (*b* = 0.327, *p* < 0.001), and perceived unfairness is negatively and significantly related to voice behavior (*b* = −0.297, *p* < 0.001) and job performance (*b* = −0.283, *p* < 0.001). Thus, Hypotheses 3, 4a, and 4b are supported by the data. Meanwhile, the moderating effect of credit-claiming attribution to protect employees is significant, as the *p*-value of each relationship (*b* = −0.139, *p* < 0.001; *b* = −0.361, *p* < 0.001) is greater than 0.05.

**TABLE 5:** Hypothesis testing.

Effect	Hypothesis	Path coefficient	*t*-test	*p*-value
Main effect	Hypothesis 1	0.439	10.309	<0.001
	Hypothesis 2a	–0.321	7.496	<0.001
	Hypothesis 2b	–0.306	6.854	<0.001
	Hypothesis 3	0.327	8.193	<0.001
	Hypothesis 4a	–0.297	6.562	<0.001
	Hypothesis 4b	–0.283	6.017	<0.001
Moderating effect	Hypothesis 5a	–0.139	3.262	<0.001
	Hypothesis 5b	–0.361	11.027	<0.001

**FIGURE 2:**
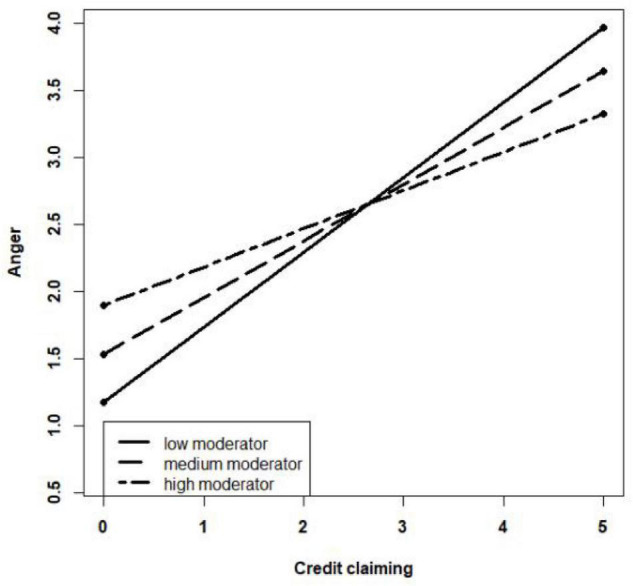
Moderating effect of credit-claiming attribution to protect employees on the relationship between leaders’ credit claiming and employees’ anger.

**FIGURE 3:**
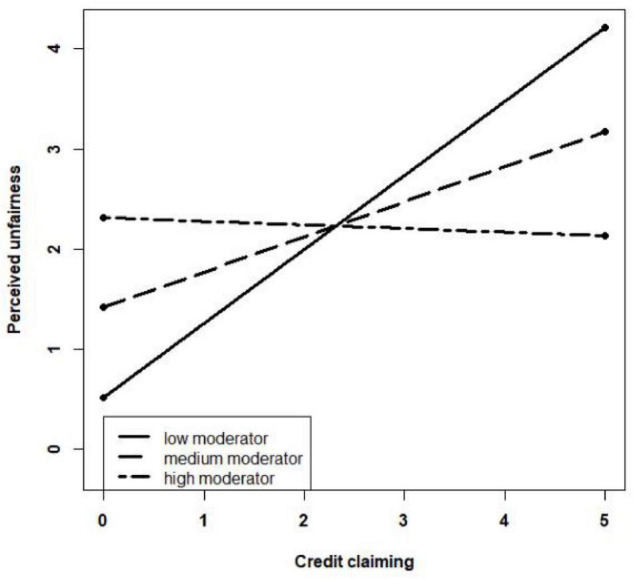
Moderating effect of credit-claiming attribution to protect employees on the relationship between leaders’ credit claiming and employees’ perceived unfairness.

As the path coefficients of the moderating effect are significant, *R* is used in the simple slope test to investigate further the moderating effect of credit-claiming attribution to protect employees on the relationship between the leaders’ credit claiming and the employees’ anger and between the leaders’ credit claiming and the employees’ perceived unfairness. The interaction mechanism diagrams are created based on the mean − SD, mean, and mean + SD of the moderator. As shown in Figure 2, when credit-claiming attribution to protect employees is high, the curve slope is slight, and the employees’ anger is less sensitive to the change in the leaders’ credit claiming. Similarly, in Figure 3, when credit-claiming attribution to protect employees is high, the curve slope is slight, and the employees’ perceived unfairness is less sensitive to the change in the leaders’ credit claiming. In addition, credit-claiming attribution to protect employees has a strong moderating effect on the relationship between the leaders’ credit claiming and the employees’ perceived unfairness because the slope changes quickly as the moderator increases. Thus, the results support Hypotheses 5a and 5b.

## Discussion

Based on affective events theory and relative deprivation theory, this study advanced and examined a model of how leaders’ credit claiming affected employees’ work outcomes (i.e., voice behavior and job performance) through the mediating role of employees’ anger and perceived unfairness and how credit-claiming attribution (i.e., to protect employees) moderated the effect of leaders’ credit claiming on employees’ anger and perceived unfairness. This research found that when leaders claimed credit for their employees’ contributions to senior managers, the employees felt anger and subjective perception of unfairness, which was not conducive to the employees’ subsequent engagement in positive in-role and extra-role behaviors at work. In addition, the results showed that the employees’ perception of credit-claiming attribution for their protection weakened the positive effect of the leaders’ credit claiming on the employees’ anger and perceived unfairness.

First, we focused on the direct relationship between leaders’ credit claiming and employees’ anger. Based on previous research (Rasool et al., 2020; Zhou et al., 2020, 2021), the results showed that leaders’ credit claiming has a positive impact on employees’ anger, which supported H1. The findings of this study were also supported by the previous studies (Pirola-Merlo et al., 2002; Cropanzano et al., 2017b). This study was also supported by the affective events and relative deprivation theory (Weiss and Cropanzano, 1996; Smith et al., 2012). However, employees feel that their leaders take their hard work or ideas without effort. This perception of their disadvantageous position triggers anger in employees. Nevicka et al. (2018) demonstrated that leaders’ credit claiming will destroy the employees’ established interests, including their sense of responsibility by contributing to the organization and satisfaction of their accomplishments, proving their abilities and value. Therefore, the above studies indicated that leaders’ credit claiming positively affects employees’ anger, which supports our findings.

Second, the findings of this study confirmed that employees’ anger has a negative impact on their voice behavior and job performance. Therefore, the outcomes are consistent with previous studies and support H2. This result is also in line with the affective events theory (Oreg and Berson, 2019). Taylor (1991) observed that adverse events prompt individuals to adjust and adapt mentally, cognitively, emotionally, and behaviorally. Therefore, anger can significantly deplete employees’ internal resources and negatively affect work outcomes (Byrne et al., 2014). Previous research found that employees’ negative emotions have a negative impact on their voice behavior (Hsiung and Tsai, 2017) and job performance (Sy et al., 2006). Similarly, the above studies indicated that employees’ anger negatively affects their voice behavior and job performance, which supports our findings.

Third, we focused on the direct relationship between leaders’ credit claiming and employees’ perceived unfairness. The results showed that leaders’ credit claiming has a positive impact on employees’ perceived unfairness, which supported H3. The findings of this study were also supported by the previous studies (Smith et al., 2012; Erdogan et al., 2018). This study was also supported by the relative deprivation theory (Xiong et al., 2021). However, leaders’ credit-claiming behavior’s hidden meaning is taking employees’ contributions for themselves without informing the employees or their consent (Weaver, 1986). Employees perceive that the organization does not recognize their contributions and efforts and that their leaders can easily reap their outcomes. This type of forced deprivation denies employees the proper and reasonable treatment for their contributions (Mauno et al., 2005). Therefore, the above studies indicated that leaders’ credit claiming positively affects employees’ perceived unfairness, which supports our findings.

Fourth, the findings of this study confirmed that employees’ perceived unfairness has a negative impact on their voice behavior and job performance. Therefore, the outcomes are consistent with previous studies and support H4. This result is also in line with the social exchange theory (Cropanzano et al., 2017a). Konovsky and Pugh (1994) observed that employees’ lack of sense of obligation and responsibility would not engage in behaviors that will help the organization operate efficiently. Therefore, perceptions of unfairness can further discourage employees from engaging in behaviors that benefit the organization. Previous research showed that employees’ perceived unfairness has a negative impact on their voice behavior (Bies and Shapiro, 1988) and job performance (Walumbwa et al., 2009). Similarly, the above studies indicated that employees’ perceived unfairness negatively affects their voice behavior and job performance, which supports our findings.

Fifthly, we tested the moderating effect of credit-claiming attribution between leaders’ credit claiming and anger and the moderating effect of credit-claiming attribution between leaders’ credit claiming and perceived unfairness. The findings of this study demonstrated that credit-claiming attribution moderates these relationships, which supports H5. Therefore, the results of this study also confirm that credit-claiming attribution to protect employees reduces the positive impact of leaders’ credit claiming on employees’ anger and perceived unfairness. These results are in line with the theory of attribution and the research of Kelley and Michela (1980). In an attribution situation, leaders claim credit to protect their employees, thereby allowing them to focus on the potential risks associated with their actions. Risks are transferred to the leaders as a result of their credit claiming. This perception of risk transfer prompts employees to appreciate the protection and support of their leaders (Detert and Burris, 2007). Therefore, the above studies indicated that in this attributional state, the positive relationship between leaders’ credit claiming and employees’ anger is weakened, and the positive relationship between leaders’ credit claiming and employees’ perceived unfairness, which supports our findings.

### Theoretical Implications

This study contributes to the literature in three ways. First, this study illustrated the detrimental effects of leaders’ credit claiming on employees’ outcomes. Previous research discussed the effect of employees’ credit-claiming behavior on organizations, such as the employees themselves and their coworkers (Maher et al., 2018; Brosy et al., 2021). However, employees may also encounter events where leaders take their contributions and claim credit to senior managers. This research observed that employees who criticize the benefits of credit claiming might experience unstable emotional conditions and a psychological imbalance. This study also determined that leaders’ credit claiming hindered employees’ positive in-role and extra-role work behaviors. Employees with poor emotional and psychological states may be reluctant to contribute to the organization. Therefore, understanding the outcomes of leaders’ credit-claiming behavior in this context is essential.

Second, this study paid attention to employees’ anger and perceived unfairness, which play a critical psychological role when leaders claim credit for their contributions. Previous research focused on the psychological state of the individual engaging in credit-claiming behavior and its changes rather than the psychological state of the individual affected by others’ credit-claiming behavior (Gerber and Gibson, 2009; Rupp et al., 2017). The present research examined whether and how leaders’ credit-claiming behavior affected employees’ emotions and psychological states and found that employees felt anger in the face of the affective event of credit claiming. The study also determined that leaders’ credit claiming can create a sense of relative deprivation and perceived unfairness in employees.

Third, this study enriched attribution research by elaborating on credit-claiming attribution as a moderating mechanism in the influence of leaders’ credit-claiming behavior on employees’ anger and perceived unfairness. Research on the psychological resource state of credit-claiming attribution is lacking (Clapham and Schwenk, 1991). Although some studies employed organizational theory to understand the outcomes of leaders’ credit-claiming behavior, research on the moderating role of credit-claiming attribution is scarce (Repenning and Sterman, 2002; Gerber and Gibson, 2009). Based on previous research on attribution, the present empirical study established a link between credit-claiming attribution, leaders’ credit-claiming behavior, and employees’ anger and perceived unfairness. This study determined that credit-claiming attribution moderated the effect of leaders’ credit-claiming behavior on employees’ anger and leaders’ credit-claiming behavior on employees’ perceived unfairness.

### Practical Implications

Our results have several practical implications. First, the findings may help improve senior managers’ understanding of credit-claiming behavior. In most cases, leaders’ credit-claiming behavior can adversely affect employees’ psychological states and behaviors and cause substantial organizational losses (Deshpande, 1997). Therefore, senior managers should take appropriate measures to reduce the negative effect of leaders’ credit claiming, such as by creating a harmonious organizational climate that encourages mutual respect, transparency, and cooperation between leaders and employees.

Second, the research findings can help leaders understand employees’ emotional and psychological states as essential elements in organizational psychology. Leaders’ credit claiming of employees’ contributions can lead to feelings of anger and fairness deprivation, which can undermine the employees’ satisfactory work outcomes in the future. Thus, leaders should deal with their relationships with employees and take the necessary steps to help them demonstrate their contributions to the organization.

Finally, the results of this study suggest that leaders’ credit-claiming attribution to protect employees can mitigate the negative impact of their credit-claiming behavior on employees’ emotional and psychological states. Previous research showed that leaders’ credit-claiming attribution to protect others is more likely to garner employees’ understanding than attribution to gain benefits (Repenning and Sterman, 2002; Detert and Burris, 2007). Therefore, leaders should provide employees with channels or platforms to communicate with colleagues up and down the hierarchy to gain their support when claiming credit for their contributions to senior managers.

### Limitations and Future Research

This study has several limitations. First, this research focuses only on the phenomenon of leaders’ credit claiming. However, leaders may adopt other relationship management forms, such as leader–employee exchanges and leadership styles. Therefore, future studies may also comprehensively examine various leader–employee relationships based on credit-claiming behavior. In addition, the data collected for this study derived from employees with Chinese collectivist culture; thus, their responses may differ from those of participants in Western cultural contexts (Hofstede, 1984). Hence, the extent to which the results of this study can be generalized to settings outside China may be limited. Future research can collect data from employees in different cultural contexts to increase the generalizability of the study.

## Conclusion

Based on affective events theory and relative deprivation theory, the outcomes of this study show the relationships between credit claiming, anger, perceived unfairness, voice behavior, job performance, and credit-claiming attribution to protect employees. Specifically, credit claiming have a direct positive impact on anger and perceived unfairness, while they have negative impacts on voice behavior and job performance. Similarly, the credit-claiming attribution to protect employees moderates the relationship between credit claiming and anger and between credit claiming and perceived unfairness.

The conclusions of this study are as follows: first, leaders’ credit claiming will have a harmful effect on the employees in the organization, and it will destroy the employees’ emotions and attitudes under such leader behavior. Second, leaders’ credit claiming damages employees’ work outcomes through anger and perceived unfairness. From the affective events and relative deprivation theories, employees perceive that they are not receiving adequate benefits for their time and effort, whereas their leaders enjoy all the fruits of their labor. Therefore, leaders’ credit claiming can further discourage employees from engaging in behaviors that benefit the organization. Finally, when encountering leaders’ credit claiming, employees establishing positive psychological attribution of leadership behavior can reduce the harmful impact of credit claiming on their anger and perceived unfairness.

## Data Availability Statement

The raw data supporting the conclusions of this article will be made available by the authors, without undue reservation.

## Ethics Statement

The studies involving human participants were reviewed and approved by the Beijing Jiaotong University. The patients/participants provided their written informed consent to participate in this study.

## Author Contributions

SC wrote theories and hypotheses. DL was responsible for data analysis. CY performed data analysis and corrected the grammar errors during the revision. XZ improved the research idea, theory, and conceptual model. LH was responsible for data collection. All authors contributed to the article and approved the submitted version.

## Conflict of Interest

The authors declare that the research was conducted in the absence of any commercial or financial relationships that could be construed as a potential conflict of interest.

## Publisher’s Note

All claims expressed in this article are solely those of the authors and do not necessarily represent those of their affiliated organizations, or those of the publisher, the editors and the reviewers. Any product that may be evaluated in this article, or claim that may be made by its manufacturer, is not guaranteed or endorsed by the publisher.
