# Supplementary material for: The Idea Is Mine! An Empirical Examination on the Effect of Leaders’ Credit Claiming on Employees’ Work Outcomes
Source: Front Psychol. 2022 Feb 18;13:818454. doi: 10.3389/fpsyg.2022.818454 (PMC8895274; doi:10.3389/fpsyg.2022.818454)
Supplement: Supplementary file 1 [file Data_Sheet_1.docx]

**Appendix**

**Credit Claiming (Proell, Sauer, & Rodgers, 2016)**

1. My leader uses my ideas without acknowledging that I came up with them.
2. My leader takes credit for my ideas.
3. My leader claims my ideas as his/her own.

**Anger (Ford, Wang, Jin, & Eisenberger, 2018)**

1. I am mad with my leader.
2. I am angry with my leader.
3. I am enraged with my leader.
4. I am furious with my leader.

**Perceived Unfairness (Grover, 1991)**

1. I feel that my leader made the wrong choice that is unfair to me.
2. I feel the result of the leader’s selection was unfair.
3. I am dissatisfied with the selection of my leader.

**Voice Behavior (Van Dyne & LePine, 1998)**

1. This employee developed and made recommendations concerning issues that affect the organization.
2. This employee spoke up and encouraged others in the organization to get involved in issues that affect the organization.
3. This employee communicated his/her opinion about issues to others in the organization even if his/her opinion was different and others in the organization disagreed with him/her.
4. This employee spoke up in the organization with ideas for new projects or changes in procedures.

**Job Performance (Tsui, Pearce, Porter, & Tripoli, 1997)**

1. This employee’s ability to perform job tasks.
2. This employee’s judgment when performing job tasks.
3. This employee’s accuracy when performing job tasks.
4. This employee’s job knowledge with reference to job tasks.
5. This employee’s creativity when performing tasks.

**Credit-claiming Attribution to Protect Employees (Yorges, Weiss, & Strickland, 1999)**

1. I perceive that my leader was acting on the basis of true beliefs to protect me in his/her credit claiming.
2. I perceive that my leader was acting on the basis of moral convictions to protect me in his/her credit claiming.
3. I perceive that my leader was acting on the basis of beliefs about potential rewards for his/her own benefit in the credit claiming.
4. I perceive that my leader was acting on the basis of external pressures for his/her own benefit in the credit claiming.
